# A taxonomy and rating system to measure situation awareness in resuscitation teams

**DOI:** 10.1371/journal.pone.0196825

**Published:** 2018-05-14

**Authors:** Thomas A. O’Neill, Jesse White, Nicole Delaloye, Elaine Gilfoyle

**Affiliations:** 1 Department of Psychology, University of Calgary, Calgary, Alberta, Canada; 2 Faculty of Law, University of Toronto, Toronto, Ontario, Canada; 3 Department of Pediatrics, University of Calgary, Calgary, Alberta, Canada; University of Cincinnati, UNITED STATES

## Abstract

Team SA involves a common perspective between two or more individuals regarding current environmental events, their meaning, and projected future status. Team SA has been theorized to be important for resuscitation team effectiveness. Accordingly, multidimensional frameworks of observable behaviors relevant to resuscitation teams are needed to understand more deeply the nature of team SA, its implications for team effectiveness, and whether it can be trained. A seven-dimension team resuscitation SA framework was developed following a literature review and consensus process using a modified Delphi approach with a group of content experts. We applied a pre-post design within a day-long team training program involving four video-recorded simulated resuscitation events and 42 teams across Canada. The first and fourth events represented “pre” and “post” training events, respectively. Teams were scored on SA five times within each 15-minute event. Distractions were introduced to investigate whether SA scores would be affected. The current study provides initial construct validity evidence for a new measure of SA and explicates SA's role in resuscitation teams.

## Introduction

Evidence-based training and guidelines are in place to support rapid decision-making under various emergency clinical circumstances such as resuscitation [1, 2]. However, it has been noted that performance is highly dependent on “non-technical” or “teamwork” aspects of performance, such as communication, leadership, decision-making, and situation awareness (SA) [3, 4]. SA in particular appears to be a critical requirement for effective responses and performance in high-pressure, dynamic environments such as critical care, emergency, and resuscitation [5–8]. As these emergency situations are dealt with through teamwork, *team* SA is of particular importance [9].

In the current study we offer several contributions to the existing literature on team SA. First, we develop a framework of team SA for emergency situations. This is important because while SA is multidimensional, the dimensions of team SA for emergency situations remain unclear. Furthermore, dimensions of SA are needed for precision measurement. Second, we advance a measurement system that uses unobtrusive observer ratings. This fills an existing gap because SA has traditionally been measured by freezing scenarios and questioning team members [10]. That methodology is unsuitable when freezing scenarios is impossible (e.g., when observing real resuscitation events). Thus, we offer reliability evidence that captures the dynamics of team SA over time. Third, we report on construct validity evidence using three approaches: correlations with overall team performance, change as a result of a distraction event, and change associated with team training (using pre/post methodology). This deepens existing theoretical understanding of the role of SA in teamwork during emergency situations and sets the stage for future research using a common framework and measurement standard.

### Team situation awareness

Individual SA refers to one’s “awareness and understanding of the dynamic information that is relevant to the current environment and task”(p. 97) [11]. Schulz et al. [8] analyzed 200 critical incidents in anesthesia and critical care, and found that SA-related errors were present in 81.5% of cases. Errors of perception were most frequent (38%), followed by comprehension (31.5%) and projection of future events (12%). Clearly, SA is critical to individual performance and error reduction in emergency situations.

Salas et al. [12] noted that team SA is also critical to team performance due to “unique activities, such as coordination and information sharing” (p. 126). Consistent with this, we adopted a process view of SA [13] and investigated it as a team process through which team inputs (e.g., novel information) could be transmitted to team outputs (e.g., patient survival). This fits with the classic input-process-output (IPO) model of team effectiveness [14]. Under this framework, SA can be assessed at the team-level through behavioral indicators displayed by team members with respect to dimensions of team SA [12].

### Measurement of team SA for observation of real-world emergency situations

The most common approach to measure SA is the freezing technique, in which a scenario is paused and team members are asked about their SA before the scenario resumes [7, 15, 16]. In some situations, however, the freezing approach is inappropriate. First, as the current study took place within a team training context, it would have adversely affected the learning experience and realism to interfere with the resuscitation team simulations repeatedly, as Endsley’s [10, 15] method requires. Second, freezing scenarios would have interfered with the collection of other performance outcomes, such as time to completion of key clinical tasks. Third, the freezing technique is impossible to apply during actual resuscitation and critical care emergency events. Given that the field needs to move in the direction of studying behavior in real situations rather than simulations [17], there is a need for an unobtrusive observer rating system that is multidimensional, reliable, and valid. Importantly, a recent systematic review did not find any unobtrusive measures developed specifically for team SA [18].

#### Study objectives

We had three objectives for our current research. First, there is a need to identify a multidimensional taxonomy that captures the complexity of team SA in behavioral terms. Although the multidimensionality of team SA is widely acknowledged [19], we did not find any investigations into a comprehensive set of behavioral dimensions suitable for resuscitation situations. Creating a taxonomy capturing the multidimensional structure of SA provides a nuanced understanding and supports highly precise and accurate measurement of specific behaviors [20].

Second, measurement of SA must be reliable despite its dynamics over time [21, 22]. It is important to consider three temporal features for SA: (a) establishment at the beginning of a task; (b) maintenance during the task, especially if it is threatened by a disruption or distraction; and (c) recovery or reestablishment after degradation [23]. Although various overall teamwork rating scales have been developed for a variety of healthcare environments, such as surgery [4], anesthesia [24], and resuscitation [25], they do not emphasize SA specifically nor are they useful to describe changes in performance over time. Rather, they use a single global rating of SA as part of a larger clinical teamwork assessment for an entire event. We aimed to capture the dynamics of teamwork through repeated, unobtrusive measurements.

Third, investigation of construct validity is critical to ensure that the inferences drawn from the assessment scores are accurate [26]. To investigate construct validity, we examined criterion validity by correlating the team SA scores with a measure of overall team performance (i.e., the Clinical Teamwork Scale; CTS) [27]. As team SA is thought to play a vital role in team performance [28], a significant correlation would support the criterion validity of the new measure. We also report on change scores resulting from study manipulations. Specifically, we conducted resuscitation simulations in which a distraction was introduced to assess whether SA levels would decline as a result of the distraction [29]. Finally, we report on a pre-post simulation-based training design aimed at enhancing SA in resuscitation teams. If post-training SA scores are higher, this supports the validity of the new SA framework and tool [30].

## Method

### Objective 1: Development of an SA taxonomy through content validity

Development of a team SA taxonomy was approached within a broader investigation of teamwork behaviors needed for effective resuscitation [31]. A literature review was conducted in order to develop an exhaustive list of teamwork behaviors from which to develop the team SA framework for resuscitation. The searches were conducted on March 22, 2010 and included PubMed, EMBASE, Eric, Google Scholar, and Web of Science databases (S1 File contains additional search details and results). Search terms included patient care team; leadership/education; cardiopulmonary resuscitation/education; crisis resource management; education; teamwork; medical education; training; staff training; resuscitation; and team. There were no formal inclusion or exclusion criteria. Articles were scanned for relevant teamwork behaviors. After removing duplicates, 48 unique behaviors were identified as a starting point for a team SA framework. Next, a 13-member expert panel was struck, which included resuscitation researchers, clinicians, and educators from medicine, nursing, and respiratory therapy across Canada. Panel members participated in a two-round modified Delphi process [32–34] in order to achieve consensus on the final list of behaviors for the resuscitation team framework. Panel members rated the extent to which each behavior should be included in the final framework using a 5-point Likert scale, ranging from “strongly disagree” to “strongly agree”. They were also asked to classify each behavior in terms of 4 main teamwork concepts: SA, role responsibility, communication, and decision-making [31]. It was determined *a priori* that an item would be included if more than 70% of the panel members rated that item either “agree” or “strongly agree.”

Round 2 was similarly structured. Next to each behavior on the ranking form was a summary of the number of panel members who ranked each item on each point on the Likert scale during Round 1. After Round 2, the original list was narrowed down to 19 unique teamwork behaviors. Of these behaviors, seven were classified as team SA behaviors by the panel. See S1 File for identification of linkages between our dimensions and previous research in the field. See S1 File a section mapping this taxonomy to Endsley’s [15] three-dimensional model, and we find strong construct correspondence and content validity support.

### Objective 2: Development of a reliable SA assessment

Each dimension in our SA framework represents observable behaviors from which varying levels of SA can be scored by trained observers. A scoring key (Table 2) and example behaviors (Table 3 and S1 File) were developed for the SA assessment based on a subset of teams detailed below (see also *Measures*, below). For example, reassessing a patient’s vital signs allows the team to be aware of changes to the current situation, thereby supporting team SA (and resulting in positive points). In contrast, when a team leader fails to prioritize attention to the patient by attending to a phone call, team SA is reduced (resulting in negative points). A zero score was also needed in cases when the behavior was absent. Not all dimensions have a relevant behavior that could be described as supporting or diminishing SA; therefore not all dimensions have both positive and negative scoring options. Dimension scores were also truncated at particular values. For example, if a team has already reassessed a patient three times, an additional reassessment would not add further to team SA. Therefore the scoring key was truncated at three in this case. This scoring methodology was created by observing and coding a subset of teams not included in the main study (see below). Further details are provided in the S1 File.

### Objective 3: Construct validity of the new SA assessment

#### Participants

Study participants represented all clinical professions normally present on a resuscitation team: physicians, nurse practitioners (NP), registered nurses (RN), and registered respiratory therapists (RRT). The study comprised 300 clinicians in 51 teams; however, data for 9 teams was unusable, leaving data from 42 teams and 242 participants for final analysis (81 physicians, 2 NPs, 105 RNs, and 54 RRTs).

#### Intervention

A one-day simulation-based training intervention was developed and pilot-tested with pediatric resuscitation teams at a large Canadian university [31]. Following the pilot study, a multi-center evaluation of the training was conducted [47]. This phase of the study took place at four university-affiliated children’s hospitals across Canada. All centers obtained Institutional Review Board approval and all participants provided voluntary, written informed consent prior to participation. The Board approvals were obtained from: Conjoint Health Research Ethics Board from the University of Calgary, Sick Kids Research Ethics Board, University of Alberta Research Ethics Board, McGill University Health Centre Research Ethics Board. All participants were adults.

The simulation-based team training intervention consisted of an interactive lecture on teamwork content (including SA), group review of video-recorded examples of effective and ineffective team performance and SA, and participation in four 15-minute simulated pediatric resuscitation events followed by facilitated group debriefs [47] (Table 4). The teamwork content consisted of 16 desirable behaviors related to communication, role clarity, and decision-making. The SA content consisted of seven desirable dimensions (described within our taxonomy; see Table 1). Of the four resuscitation events (detailed in Table 4), the first (referred to as the “PRE” scenario) and fourth (referred to as the “POST” scenario) used the same progression of clinical events (cardiogenic shock leading to ventricular tachycardia), but with modifications to the patient history between the two. We use the term PRE because this scenario occurred prior to presentation of teamwork and SA content; therefore it represents the teams’ baseline achievement. We use the term POST because this was the final simulation of the day-long training and it was equivalent to the PRE event, which allowed for pre-post comparisons. The second and third scenarios were randomly assigned to be either the SVT or asystole scenario (see Table 4).

**Table 1:** Proposed seven-dimensional SA framework and sample sources.

SA Dimension	Description	Relevant Sources
1. Allocate resources	Team efficiently uses all members to accomplish tasks. Necessary equipment is quickly retrieved	[20, 24, 35–40, 42, 43]
2. Anticipate and plan	Team members make projections about the future: what patients don’t need right now but may need down the road, so preparations can begin	[20, 24, 37–42]
3. Avoid fixation errors	Team members ensure that they don’t fail to use all information to revise diagnosis or plan if needed	[35, 36, 38, 40, 42]
4. Call for help when needed	Awareness that the team does not have the expertise necessary to handle the current situation so they need support from others	[35–40]
5. Prioritize attention	With many pieces of information available at once, team members decide on what to focus on at any given moment, which will change over time	[37–40, 42]
6. Reassess patient	Allows team members to be aware of changes in the patient’s clinical status so decisions can be made about diagnosis and therapy	[24, 35, 38, 40]
7. Shared mental model	Team members are all up to date on what has happened, what is happening, and what is going to happen	[41–46]

**Table 2:** The SA assessment rating format for time 1 (other time points are identical).

Interval	SA Dimension	Score				
0:01–3:00 Minutes	Allocate resources	-2	-1	0	1	2
	Anticipate and plan	-2	-1	1	2	3
	Avoid fixation errors	-2	-1	0	1	2
	Call for help when needed		-1	0	1	
	Prioritize attention	-2	-1	0		
	Reassess patient			1	2	3
	Shared mental model			1	2	3

**Table 3:** Example behaviors scored on dimensions of SA.

SA Dimension	Example Behaviors	
	Behaviors Supporting SA	Behaviors Diminishing SA
Allocate Resources	• Assigns all members on a task • Switching of members’ tasks when tired or need to leave • Direction from leader to *specific* person	• “Up in the air” communication, vague directions from leader
Anticipate and Plan	• Projects (vocalizes) future possibilities • “If/then” statements (“If the patient goes apnic, then we will need to intubate”) • Prepares medication / equipment *before* necessary	• Having to prepare medication / equipment after starting procedure
Avoid Fixation Errors	• Suggests possible alternative diagnosis • Acknowledges inconsistent information (non-response of bolus) • Makes use of new information to consider other routes	• Giving a third fluid bolus (despite not having beneficial effect) • Diagnosis of “sepsis” (despite availability of alternative) • Treating other symptoms while patient needing shock
Call for Help When Needed	• Calls cardiology • Calls for help from ICU	• Failure to call cardiology, despite apparent heart problem
Prioritize Attention		• Leader takes a “hands-on” approach • Leader answers phone / attends to distracting confederate
Reassess Patient	• Asks for a “head-to-toe” or ABCs • Asks for pulse check • Asks for update on blood pressure	
Shared Mental Model	• Recap of code history so far • Vocalizes possible diagnosis	

**Table 4:** Sequence of events in resuscitation scenarios and event details.

Clinical context of scenario	Clinical events	Distractions by confederate actors
Cardiogenic shock (run first as “PRE” and last as “POST” scenarios)	Event 1 (initial state): patient tachycardic and poorly perfused with low blood pressure Event 2: improves with fluid resuscitation Event 3: deteriorates with further fluid resuscitation Event 4: develops pulseless ventricular tachycardia (VT)	At exact time of pulseless VT, team leader interrupted to provide results from previously performed blood test or chest x-ray
Supraventricular tachycardia (SVT)	Event 1 (initial state): patient in unstable SVT, requiring cardioversion Event 2: briefly converts back to sinus rhythm after cardioversion but returns back into unstable SVT	Cardiologist enters scenario and orders an antiarrhythmic drug. The order is deliberately incorrect.
Asystole	Event 1 (initial state): patient is apneic and in asystole Event 2: after epinephrine is given, patient has return of spontaneous circulation	Parent is emotional and repeatedly asks team members for update on sequence of events.

*Note*. We standardized the order of the four resuscitation events throughout the day-long training. The second and third trials were randomly assigned; however, the ordering of those scenarios would have no bearing or carry-over effects on the fourth scenario (i.e., POST).

Each simulated resuscitation event was conducted using the Laerdal SimBaby™ high-fidelity mannequin. Setup of the room, equipment available, and video camera angles were standardized across the four participating centers. Team members were instructed to participate in the event as they normally would in real life, including assuming roles similar to what they would normally be assigned, calling for help, and using standard equipment. Progression of clinical events within each simulated resuscitation event was standardized as well, including detailed instructions for confederates (actors) regarding how he/she was to play his/her role with the team. Importantly, 10 minutes into both the PRE and the POST simulation, a distracting event was planned concurrent with a key clinical transition point (see Table 4). A confederate either entered the scenario or called into the room attempting to distract the team leader with a test result while the patient transitioned into ventricular tachycardia. The distraction challenged teams to maintain SA in a dynamic environment and to recover SA afterward.

#### Measures

A sample of six videos of simulations (not included in the final sample) was reviewed to determine behaviors that would be indicative of each dimension of team SA and to set the ranges in the scoring key. As discussed in more detail in the S1 File, dimensions were scored on a scale ranging form -2 to +3 (anticipate and plan); -2 to +2 (avoid fixation errors), +1 to +3 (shared mental model), or based on a count with penalties or gains (calls for help when needed). Here we note that different scoring was used because some dimensions are best rated as an overall effectiveness within a range that includes (avoid fixation errors) or does not include zero (anticipate and plan); because it could only be to some degree present (shared mental model); or because it could easily be counted as having happened or should have happened (calls for help when needed). Each 15-minute scenario was divided into five three-minute intervals so that SA could be measured dynamically. While developing the scoring key, we found that three-minute increments were important for several reasons. First, a time span that is too short leaves insufficient opportunities to display behaviors related to team SA. Second, a time span that is too long can lose the opportunity to capture changes in SA over time. Moreover, it can result in too much behavioral variability within a time period to provide a stable point estimate of team SA at a given time. Third, the distraction was applied at exactly the 10-minute mark of the simulation, and therefore the time intervals needed to align with this such that the distraction was near, but not exactly at the beginning, of a given time period.

Two raters were trained on the use of the SA assessment tool in order to assess inter-rater reliability. One coder scored all videos and a second coder scored a random subset of 10 videos. Videos were scored on the team SA scoring sheet (see Table 2). Within each three-minute interval, teams received a score for each of the seven dimensions that were then summed to form an aggregated SA score for each time interval. SA scores for each interval were also summed into an aggregate SA score for the entire simulation (see also S1 File).

Independent from the SA scoring described above, team performance was assessed using an overall teamwork assessment tool (i.e., the CTS) [27]. The CTS follows a common approach to assessing teamwork performance in resuscitation where various teamwork competencies are all measured simultaneously and combined to describe overall team performance [24, 48, 49]. The CTS including ratings for four teamwork domains: role responsibility, communication, SA, and decision-making. Fourteen video reviewers, who were independent from the two coders involved in scoring SA, were trained to rate resuscitation team performance [47]. Two reviewers were randomly assigned to review each video from the larger population of 14 reviewers, ensuring that the reviewers were always blinded to the participants (i.e., the reviewer did not work in the same center as the participants) and the conditions (i.e., PRE or POST).

For researchers interested in conducting a direct replication of this research (rather than a conceptual replication, which should be possible given the methodology described here), a team member from our research team would be willing to visit the site and conduct a “train the trainer” workshop.

#### Empirical analysis

Inter-rater reliability for the team SA measure was examined by reporting the zero-order correlation involving ratings from the two trained coders. To assess criterion validity (correlations of team SA with a team outcome), we report the correlations involving team SA and scores on the CTS (each was scored by unique coders that were not in contact). Because there were more than two trained coders using the CTS, we report the intra-class correlation (ICC) as a measure of inter-rater reliability. Finally, the full design involved a 2 x 5 ([PRE, POST] x time interval) fully-crossed repeated measures analysis of variance (RANOVA). The design is fully-crossed and repeated because all teams were in both the PRE and POST conditions and all time intervals. This design allows us to investigate PRE versus POST scores, as well as the effect due to time and the interaction.

## Results

### Objective 1: Development of a team SA taxonomy

The SA taxonomy contains the dimensions in Tables 2 and 3 as identified in the Method and S1 File. For consideration of how the current taxonomy maps to Endsley’s (1999) work, please refer to the S1 File.

### Objective 2: Development of a reliable team SA assessment

We computed correlations between raters’ scores of SA within videos across time as a measure of inter-rater reliability (*n* = 10 for the subset of videos coded by both trained raters). Inter-rater reliability was in the acceptable range at all time points (Time 1 = .89, *p* < .05, Time 2 = .86, *p* < .05, Time 3 = .83, *p* < .05, Time 4 = .77, *p* < .05, and Time 5 = .86, *p* < .05). The aggregate SA scores averaged across time were highly reliable (*r* = .96, *p* < .01).

### Objective 3: Construct validity of new SA assessment

As a measure of criterion validity, CTS scores were considered as a possible correlate of the new team SA measure. We correlated the SA score with the overall CTS score, because we are assessing the criterion validity of the SA measure (not the convergent validity). Criterion validity involves assessing whether a target measure is related to a theoretically-relevant outcome [50]. It is important because SA is theoretically linked to teamwork performance, and the absence of an empirical linkage would raise concerns about the validity of the SA scores. We did not focus on the CTS SA rating as a measure of *convergent* validity because theory suggests that team SA is multidimensional and dynamic, thereby requiring multiple behavioral dimensions and measurements over time, which the CTS does not permit. The CTS is, however, a standard assessment of overall teamwork performance.

**Fig 1:**
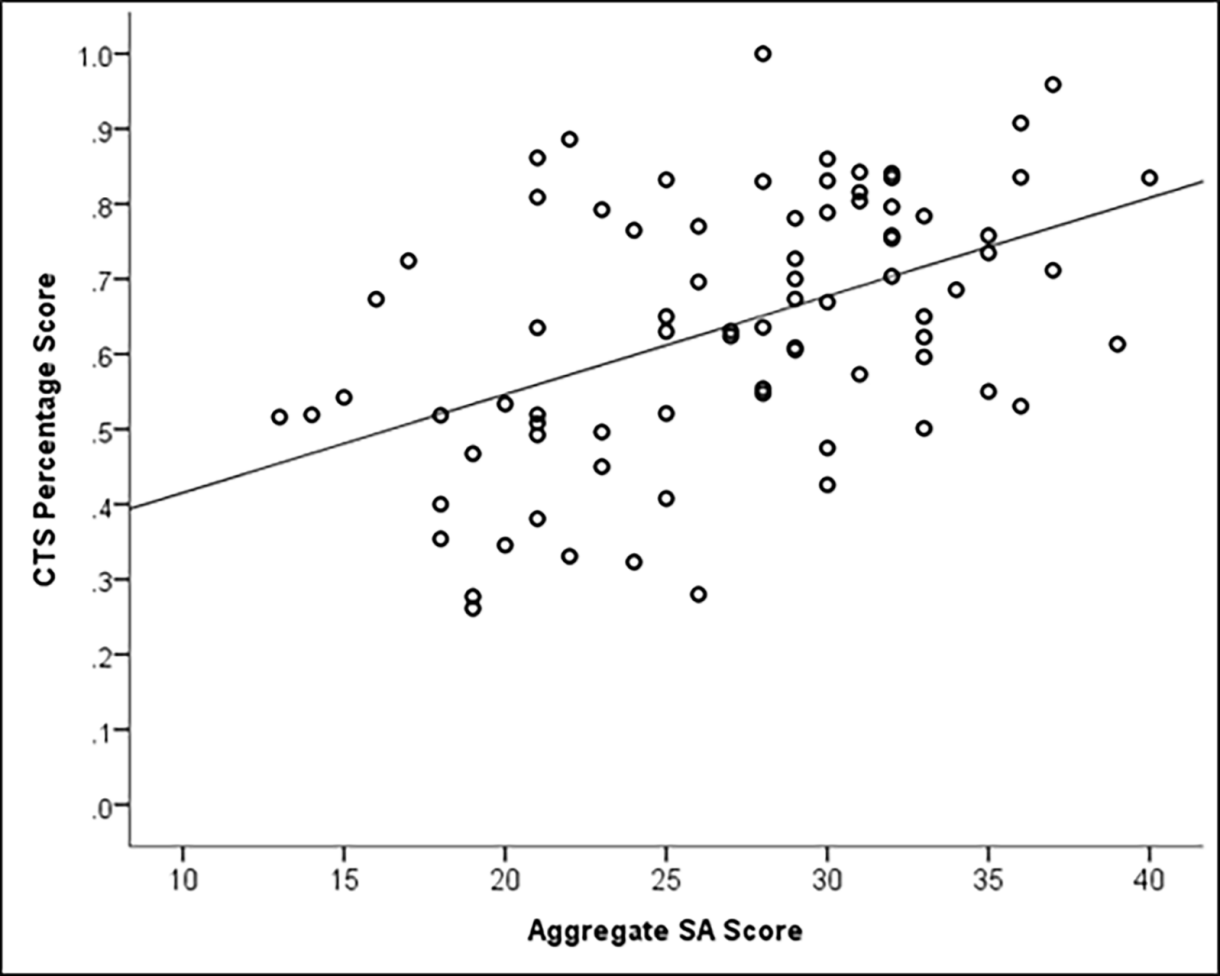
Scatterplot summarizing the relationship between SA and CTS percentage scores. The solid line represents the line of best fit. CTS = Clinical Teamwork Scale; SA = Situation Awareness.

We applied the (ICC) to assess inter-rater reliability of the CTS scores (ICC = .61, *p* < .01) [47]. Supporting the construct validity of the new SA measure, there was a moderate to strong positive correlation between SA and CTS scores (*r* = .47, *p* < .01; see Fig 1).

**Fig 2:**
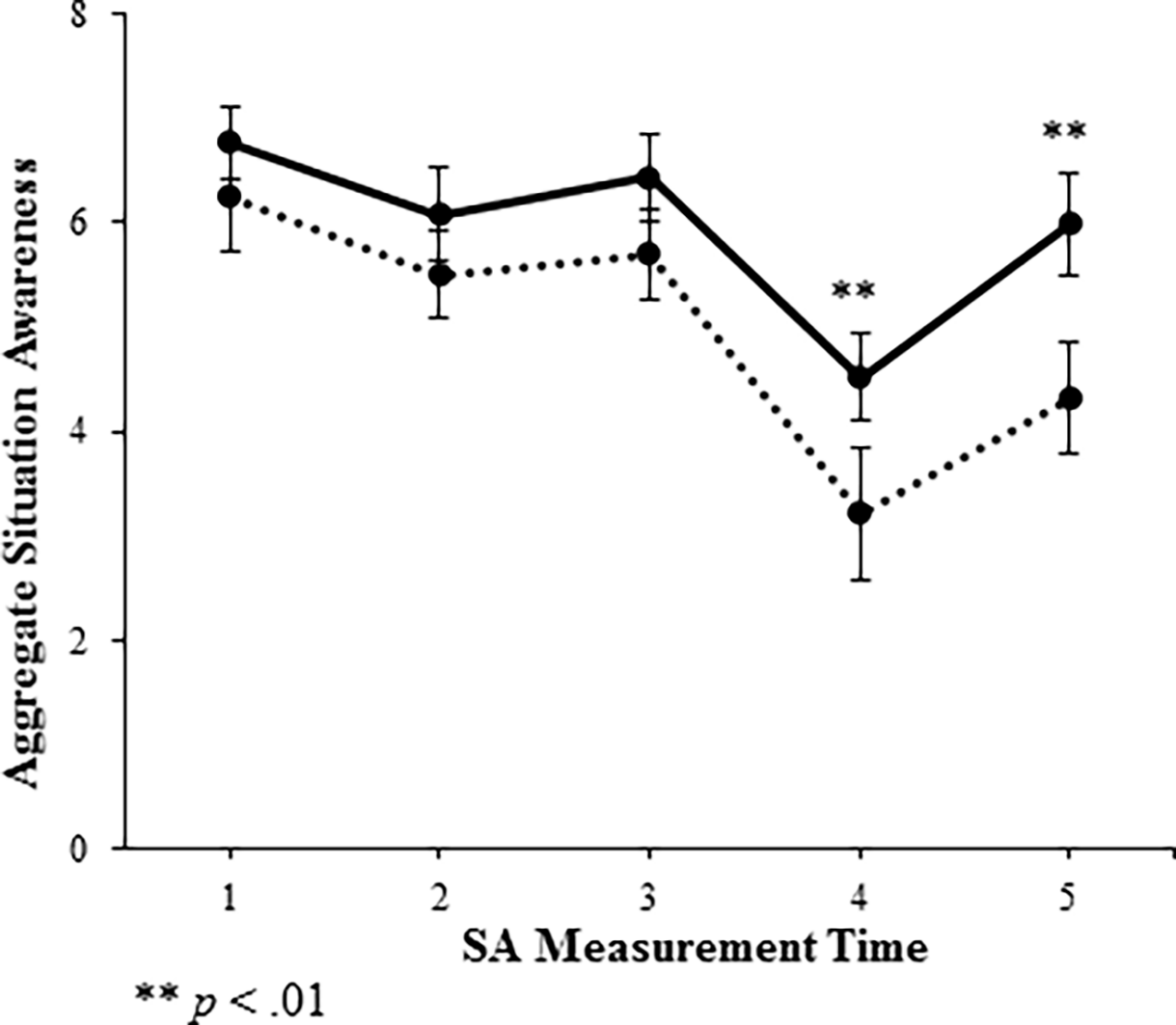
Aggregate SA scores across measurement intervals for PRE and POST scenarios. Dotted line is PRE, Solid line is POST.

A 2 x 5 ([PRE, POST] x time interval) fully-crossed RANOVA was conducted with the SA scores as the dependent variable. This analysis revealed the expected main effect for PRE-POST scores given that scores were significantly higher for the POST (*M* = 29.76, *SD* = 4.80) compared to the PRE scenario (*M* = 24.95, *SD* = 6.77, *F*(1, 41) = 24.34, *p* < .01). With respect to the distractions, collapsing across training conditions, paired *t*-tests indicated that the mean at Time 4 (*M* = 3.87) score was significantly lower than means at all other time points (Time 1 = 6.50, Time 2 = 5.79, Time 3 = 6.06, and Time 5 = 5.14, all *p* < .01), thereby supporting the team SA measure’s ability to detect changes associated with the distraction that was present during the scenario at Time 4 (10 minutes). Importantly, the RANOVA also revealed a significant interaction between scenario (PRE, POST) and measurement time, *F*(4, 38) = 3.76, *p* = .01, indicating that the difference between PRE and POST SA scores depended on when in the simulation the SA measurement was taken. Paired *t*-tests investigating the interaction revealed that at Time 4 SA scores in the POST scenario (*M* = 4.52, *SD* = 1.44) were significantly higher than in the PRE scenario (*M* = 3.21, *SD* = 2.09), *t*(41) = 3.42, *p* < 0.01, indicating a greater decrement to SA in the PRE scenario. Similarly, at Time 5 teams had significantly higher SA in the POST scenario (*M* = 5.98, *SD* = 1.62) compared to the PRE scenario (*M* = 4.31, *SD* = 1.72), *t*(41) = 5.66, *p* < .01, indicating a prolonged recovery period in the PRE scenario. PRE and POST scores were not significantly different at the three times preceding the distraction (see Fig 2).

## Discussion

In the current study we advance a new taxonomy of team SA for emergency situations and we provide evidence regarding its reliability and validity. The measure is multidimensional, reliable, unobtrusive, captures the dynamic nature of SA, and was influenced by team training and distractions in the expected pattern. We adopted a process perspective [51], which fits well with existing theories of teamwork [14]. Below we examine the implications for research and practice.

### Implications for research and practice

Many authors have published frameworks describing effective teamwork in healthcare [3, 44], yet a multidimensional taxonomy and unobtrusive rating system for the measurement of team SA in resuscitation is absent [18]. In this research, we advance a taxonomy for resuscitation situations in order to delineate the multiple facets of team SA (Objective 1) through content validity methodology. We found that resuscitation team SA is multidimensional and involves (a) calling for help when needed, (b) reassessing the patient, (c) anticipation and planning, (d) shared mental models, (e) prioritizing attention, (f) avoiding fixation errors, and (g) resource allocation. This is aligned with other behavioral approaches used to measure SA in previous studies in military settings [52–54]. The indicators we identified as important for each dimension of SA ultimately shed light on the behaviors that characterize scoring levels on these dimensions. Thus, training and education programs could utilize this taxonomy for both teaching and assessment purposes.

Our goal was to identify dimensions of SA and connect them to existing models for generalizability purposes [12, 28, 55], but also operationalize them appropriately for emergency situations. Sarter and Woods [21] noted the inevitable tension between general models of SA and the specific features of situations and tasks. Thus, whereas a common core of dimensions might be achievable, the operationalization of these dimensions will depend on context. In terms of how far we believe the current taxonomy could be generalized, we invoked Cooper et al.’s [18] definition of emergency situations as “acute medical events occurring in any clinical or simulated setting” (p. 2).

A potential concern for many behavioral rating systems is that subjective ratings are prone to error [12]. Indeed, this was a problem noted by Cooper et al. [18] in their review of current unobtrusive, behavioral measures of team effectiveness that contained SA as a subdimension. The strong inter-rater reliability observed in this study is a unique strength of the new tool (Objective 2). With reliable scoring it is now possible to examine teams’ SA over time as well as how SA relates to performance and how it may be affected by events and training. This is particularly noteworthy as the field moves toward the use of video review of actual resuscitation situations, which is critical to identify the role of teamwork factors such as SA as real events unfold and for future training purposes. Importantly, we would advise against using the rating system for real-time measurement of team SA without further study. The reliability and validity has not been investigated in this context and is therefore unknown. Our goal was to develop an observer rating system that would allow us to code actual resuscitation events that were video-recorded (see below), as video-recording of resuscitation events is becoming increasingly common.

We found considerable evidence in support of the validity of the new SA measure (Objective 3). The results indicate that the assessment has solid criterion validity as demonstrated by the moderate to strong correlation of the aggregate SA scores with the CTS [56]. Furthermore, if the new measure is accurately capturing team SA, we would expect to see a decline during a distracting event, as attention would be temporarily directed toward the distraction rather than the relevant task [23]. This expected decline was detected, which supports existing theorizing that distractions can lower team SA [23]. Given that SA is important for team performance and clinical decision-making [11], distractions must be managed carefully in any emergency situation. Future research should consider, for example, whether policy decisions to keep family members in the room during resuscitation affects team performance through distraction.

The differences between SA scores in PRE and POST scenarios provides additional insight into construct validity. The results indicate that team training has a significant effect on teams’ ability to maintain SA over the course of a task, which corresponds well with results from meta-analyses of team training [45]. Interestingly, training did not have a significant effect on teams’ ability to establish SA at the beginning of the task. This finding may suggest that medical teams already have an intuitive ability for establishing SA, and therefore team training is more beneficial for improving the ability to maintain awareness when distractions threaten SA. Moreover, trained teams not only maintained higher SA during the distraction event, but they also recovered faster than untrained teams following the event. These results indicate that training and distraction management may be crucial to maintaining high team SA. Team training programs targeting team SA should continue to be widely implemented.

Based on the above evidence, we are confident that the findings will hold and that we are, in fact, measuring team SA. In addition to the study results, we developed the SA measure using a deductive, rational test construction [57] approach following best practices and using content validity [58]. Specifically, we engaged experts in a multi-stage Delphi process to establish and refine the content of team SA within resuscitation contexts. Having identified the domain of SA based on expert knowledge, we identified behaviorally-relevant cues to serve as indicators of team SA (i.e., the team SA measure) in an initial subsample. Thus, we identified the content of the target construct and subsequently aligned the behavioral measurement to that content through observation and testing.

Given that distractions are an inevitable component of the resuscitation environment, it is vital that teams are adequately trained to deal with them. Distractions can cause delays in delivery of vital therapies to patients in cardiac arrest, which impacts survival [59]. Our novel team SA measure will be useful for assessment of team SA as well as the instruction and evaluation used in team training interventions. It is uniquely well equipped to handle the measurement of team SA when it is impossible to use the freezing technique. As future research needs to consider real resuscitation events as they unfold in order to develop a stronger understanding of how team SA relates to actual patient outcomes, video-recorded resuscitation events should be coded and used in subsequent training as well as informing policy-related decisions regarding potential distractions such as family presence.

A limitation of using observer ratings is that it is impossible to confirm SA knowledge unless it is acted upon, and even when SA behaviors occur we can only infer indirectly that it is the result of cognitive, knowledge-based SA [13, 28]. On the other hand, even if a team has high cognitive SA, if they do not act on that information their SA is not helpful. Therefore, we believe the current behavioral measure is advantageous in certain situations.

A limitation to this study is that the pre-post design lacks a control group; therefore, we cannot be certain that the training caused the stronger levels of team SA. A suitable control group would be another set of teams that participated in the simulations but did not receive the training. If the teams in the training group scored higher on team SA in the final simulation that did the teams in the control group, and the study used random assignment, we could be more confident that training causally increased team SA scores. A control group was not feasible in the current study as it took place within a training program. Thus, future research should include a control group in the research design in order to establish causality.

Future research should also investigate how the team SA dimensions advanced here load onto the larger team SA construct. Specifically, we developed a scoring rubric for each individual team SA dimension. The raw scores can be summed to arrive at a total team SA estimate of the construct-level score. However, to the extent that these dimensions tend to have different score levels (e.g., by virtue of some scales using a truncated range of score levels), the dimensions will have “de facto” weightings. In addition, this scoring approach assumes that a “-2” in anticipate and plan is more harmful than a "-1" in calls for help although this is an assumption of the scoring. We recommend factor analyses that can identify weights indicating the extent to which each dimension loads onto the team SA factor. These weights could be used to adjust the dimensions in a linear aggregate estimate of the team SA score. Moreover, if the weights are based on standardized scores, then if applied to standardized dimension scores in aggregation there will be less of an influence of truncating the scores on some dimensions.

Finally, it is worth noting that the literature review (see Method and S1 File) is a little out-dated now. It was completed prior to beginning the large training study reported on here, and therefore there has been a lag involving the original literature review used to develop the framework. Future research should consider incorporating more recent work.

### Conclusion

In the current research we developed a taxonomy of team SA as well as a reliable and valid rating scale for unobtrusive, observer-based measurement using video review. Based on our evidence, the new scale should be useful for the study and training of team SA in emergency situations when interruptions involving the freezing technique are impossible or undesirable.

## Supporting information

S1 File Additional details of literature review, scoring, and mapping to Endsley’s dimensions. (DOC)
