# Supplementary material for: A taxonomy and rating system to measure situation awareness in resuscitation teams
Source: PLoS One. 2018 May 14;13(5):e0196825. doi: 10.1371/journal.pone.0196825 (PMC5951547; doi:10.1371/journal.pone.0196825)
Supplement: S1 File — (DOC) [file pone.0196825.s001.doc]

**Supporting Information**

**Further Details on Situation Awareness (SA) Framework and Scoring**

**Literature Search** **Details and Results**

The below table summarizes the databases, search terms, and the number of articles found and identified as relevant and unique.

| **Database/Search Engine** | **Search Terms** | **Number articles found** |
| --- | --- | --- |
| PubMed | “Patient Care Team/education” [MeSH]  “Leadership/education” [MeSH]  “Cardiopulmonary resuscitation/education” [MeSH]  “Crisis Resource Management” | - 970 articles - After reviewing title and abstracts for relevance, narrowed down to 128 articles - Most of the articles not chosen were focused on CPR education |
| EMBASE | *education/ OR  *teamwork/ OR  *medical education/ OR  *training/ OR  *staff training/ AND  *resuscitation/ | - 283 articles - After reviewing title and abstracts for relevance plus duplicates was narrowed down to 8 articles |
| Eric | resuscitation AND  education | - 133 articles - After reviewing title and abstracts for relevance plus duplicates was narrowed down to no new articles. |
| Google Scholar | team AND  resuscitation AND  education AND, all limited to “Biology, Medicine & Social Sciences” | - 12 articles - After reviewing title and abstracts for relevance plus duplicates was narrowed down to 10 new articles. |
| Web of Science | resuscitation AND  team AND  education | - 106 articles - After reviewing title and abstracts for relevance was narrowed down to 3 new articles. |

**Consistency with Previous Research**

The proposed team SA framework maps on well to concepts discussed in previous research. Shared mental models corresponds to Endsley and Jones’s [28] suggestion that team members with SA can project what other team members will do in the future based on mutual understandings of how members will behave under various circumstances [22]. Salas et al. [12] mention task allocation (maps onto our resource allocation), cross-checking (maps onto our avoiding fixation errors), coordination (maps onto our calling for help and shared mental models), and information sharing (maps onto our reassessing the patient and anticipate and plan). Responsibility involving awareness of roles and hierarchy structures proposed by Harwood et al. [60] corresponds to allocate resources and anticipate and plan. Schulz et al. [55] noted that team SA processes in anesthesiology involve self-checking (maps onto our reassess patient), coordination (maps onto our calling for help and shared mental models), prioritizing (maps onto our prioritize attention) and questioning (maps onto avoid fixation errors). Gaba et al. [61] discuss problem recognition (maps onto our reassess patients, calls for help when needed), allocation of attention, and prioritization (these two map onto prioritize attention and allocate resources) as critical SA elements. Thus, the dimensions we recovered are theoretically consistent with existing dimensions discussed elsewhere (Table 1 contains additional relevant sources supporting each dimension). In addition, we mapped the team SA framework onto Endsley’s 3 dimensional model at the end of this supplement.

**Scoring**

The scoring focused on behavioral counts in order to promote objectivity. Time intervals for scoring were set at three minutes and there were five separate time intervals (see *Method*) for each resuscitation event. When a team engaged in a positive SA behavior, the team was assigned one positive point (+1). Similarly, when a team engaged in a negative SA behavior, the team was assigned one negative point (-1). During the development of the scoring key (i.e., based on a subsample of six teams, see *Method*) it was determined that some dimensions do not have behavioral examples that enhance SA (i.e., prioritize attention) or detract from SA (i.e., re-assessment and re-evaluation, shared mental model). Thus, these dimensions only had negative or positive example behaviors, respectively. If multiple behaviors were observed within a given three-minute time interval, each behavior was coded as a +1 or -1 and then summed within the interval. Dimension scale scores had specific ranges (see scoring key in Table 2) so that teams could not receive an excess of points within a given time period (because beyond a certain point, the behavior would no longer enhance or detract from team SA, per the development of the key in pre-testing). Scores for each dimension were summed to form an aggregate SA score within each time interval.

Note that we adopted behavioral observation scales (BOS) as the basis for the scoring key as per Table 2 [50]. BOS are the most strongly supported scale format used for making frequency ratings of observable behaviors [62]. Below we describe each SA dimension and provide additional example behaviors coded as positive (+1) or negative (-1) points toward team SA for each dimension (additional examples are in Table 3).

**1. Allocate resources (human and equipment) effectively**

**Description.** Making effective use of the resources and equipment that are available to the team shows that the team has good levels of SA. Every team member should have a task to do, and they should understand and be able to execute that task.

**Behaviors.** When a team leader directs members to specific tasks, it shows that there is an awareness of who is available as well as of what needs to be done. If one member, for instance, is doing chest compressions on a patient, resources should be allocated toward replacing that individual before exhaustion sets in. Having a clear conception of how roles must be coordinated shows stronger resource allocation. Assigning the most skilled people to a task (such as assigning nurses to prepare medications) would be another example of strong resource allocation. Alternatively, certain resource allocation behaviors can be detrimental by causing confusion as to who is responsible for performing a task. This can take the form of verbal requests by the leader that are “up in the air,” a phrase used to describe directions that are not directed to a specific person. These as well as vague directions suggest poor resource allocation. Assigning a task to an individual that is already fully engaged in another important task could also create confusion.

**Scoring key.** Each positive or negative behavior in a three-minute window receives a +1 or -1, and the scale’s range is +2 to -2 overall for a particular time interval.

**2. Anticipate and plan**

**Description.** Planning for future events is required for a strong understanding of the current situation and to make projections about the future so that the teams work at maximal efficiency to accomplish all required tasks during a resuscitation event.

**Behaviors.** Anticipating that the patient may need to be intubated and asking the nurse or respiratory therapist to gather necessary equipment and medications, for instance, shows that there is an awareness of the patient’s status but also of what may come. In general, a team vocalizing future possibilities shows that they are considering future possibilities. Teams that engaged in such behavior were allotted points, whereas teams that were less prepared and more reactive (e.g., not preparing medications in advance) were penalized points. For example, it shows a lack of planning when a nurse takes extra time to draw up a second dose of the same drug he/she has already drawn up. This reflects lower SA because they need to draw up at least two doses of a drug at a time to be more efficient. Moreover, in the current simulated event, when the team realizes that the patient’s intravenous catheter is not working, if they do not ask for a replacement immediately that would indicate lower levels of anticipation and planning.

**Scoring key.** Each positive or negative behavior in a three-minute window receives a +1 or -1, and the scale’s range is +2 and -2 overall for a particular time interval.

**3. Avoid fixation errors**

**Description.** Fixation errors occur when a decision about the causes or precipitating factors within a situation is not reconsidered due to fixation on other cues. Specifically, fixation occurs when there is persistence of undesirable behaviors or courses of action despite available signals in the environment that suggest revision and taking up alternative courses of action is necessary [61].

**Behaviors.** A team that ignores vital sign information in the environment (perhaps assuming it is incorrect) and does not entertain the idea that the vital signs are accurate would be engaging in a fixation error. Specifically, within our scenario, if teams decided to give the patient multiple boluses of intravenous fluid assuming he was dehydrated (as the patient history given to them suggested) and ignored evidence that the fluid administration was not having the intended effect on the patient, they were given lower scores on this aspect of SA. Similarly, behaviors such as diagnosing the patient with sepsis (as is common in pediatric critical care) and persisting with that diagnosis despite conflicting information and cues to suggest a different diagnosis was taken as indicative of low SA. However, acknowledging inconsistent information, such as noting that a fluid bolus did not necessarily impact the patient’s vitals was considered to be behavior that showed an avoidance of fixation errors. If teams noted that they were not making the desired progress using the current diagnosis and re-evaluated the diagnosis, they were allotted points.

**Scoring key.** Each positive or negative behavior in a three-minute window receives a +1 or -1, and the scale’s range is +2 to -2 overall for a particular time interval.

**4. Call for help when needed**

**Description.** Calling for help requires that the team realize or be aware that it may not have the expertise necessary to handle the current situation, and that external input or feedback is needed (e.g., from another medical or surgical specialty).

**Behaviors.** The key behavior was considered to involve seeking consultation from experts outside the team. For example, this was observed as teams realized that the underlying issue with the patient in the simulation was a problem with his heart and consequently called cardiology for a consultation. Alternatively, the teams could realize that the problem was more severe than the current team could handle and vocalize a need to call for assistance from an attending intensive care physician, who would be an expert in leading resuscitation teams. When teams engaged in these behaviors they were deemed to be showing signs of stronger SA. Teams that failed to engage in any such behavior when appropriate were considered to be focused on other aspects of the event and therefore had a lower level of SA for the broader situation. Alternatively, if the leader or team member vocalized uncertainty as to next steps or how to do a task but failed to engage in a request for help, the team was deducted a point for this aspect of SA.

**Scoring key.** Each positive or negative behavior in a three-minute window receives a +1 or -1. The scale’s range is +1 to -1 overall for a particular time point as there was typically a maximum of one opportunity to engage in or miss an opportunity to call for help per time interval.

**5. Prioritize attention effectively as the situation requires**

**Description.** Prioritizing attention involves avoiding distractions. Distractions are factors in the environment that receive a disproportionate amount of attention relative to their importance for the patient’s outcome.

**Behaviors.** Any time the leader engages in behaviors that could have been conducted by another team member, the team loses some overall SA. Such behaviors include the leader attending to distractions during the event, such as taking phone calls, and taking a “hands-on” approach by assessing the patient him/herself or performing required tasks him/herself. Further, a team leader focused on checking for a patient’s pulse can lose awareness of other aspects of the situation. In addition, team member distractions such as dealing with a family member during an event rather than attending to the patient, or ignoring environmental cues such as alarms or monitors, detracted from team SA.

**Scoring key.** Each negative behavior in a three-minute window receives a -1, and the scale’s range is -2 to 0 for a particular time interval (attention prioritization only revealed negative behaviors). The focus was on avoiding distractions failing to attend to important environmental cues and therefore only negative points (distractions) were allocated.

**6. Reassess patient**

**Description.** Re-assessments of the patient are vital in order to monitor and act on the patient’s dynamic condition. Re-assessment of the patient involves examining the patient to determine the status of a number of potential variables.

**Behaviors.** The number of times the team leader explicitly asked for an evaluation of the patient’s status was a positive example of this dimension. Other team members could also provide status updates according to their individual assessments. Asking for the current status of the airway or breathing, checking for a pulse, or re-assessing the blood pressure were considered to be indicative of this SA item.

**Scoring key.** The scale’s range is 0 to +3 overall for a particular time interval and each positive behavior in a three-minute window receives a +1. Because each measurement of SA lasted three minutes, teams were allotted a maximum of three points on this item for each measurement. Any explicit re-assessments made beyond three in a given period were deemed to no longer be adding to the team’s overall SA. There were no re-assessment behaviors identified that would indicate lower levels of SA.

**7. Shared mental model**

**Description.** Not everyone on a team will be, or needs to be, fully aware of all aspects of the situation at all times; however, teams that are all up to date on what has happened, what is happening, and what is going to happen indicates a higher shared mental model.

**Behaviors.** Building a shared mental model is supported when the leader summarizes the case and patient status for the benefit of all team members, and when all team members hear this. Sharing the progress of the event or history of the case supports the team’s shared mental model. However, as with reassessments, there seems to be a maximum effectiveness of these behaviors in a given amount of time. If a team already shares a common understanding of the situation, then increasing the shared mental model further by re-summarizing would not be effective.

**Scoring key.** Each positive behavior in a three-minute window receives a +1 and the scale’s range is 1 to 3 (it is not possible to have a zero shared mental model in this study) for a particular time interval. Zero was not included because in our scenarios we found that a team could not meaningfully have an absence of a mental model (thus, “1” was the lowest possible score).

**Mapping to Endsley’s (e.g., 1999) 3-Stage Model**

Recall that the purpose of the new framework and coding system is to allow for the assessment of team SA when interrupting the team is not feasible. Although Endsley’s (1999) methodology works well when applied to simulations and to pose questions during “freezes” or pauses in the simulation, if a team is attempting to resuscitate an actual patient who is suffering from a cardiac arrest, it is not possible to use her technique. This presents a problem, because simulation-based team behaviors and actual team behaviors in real resuscitations may not be identical. If we want to understand team SA in actual resuscitation events, then we need an unobtrusive measurement methodology.

By using unobtrusive measures, it is impossible to directly assess cognition and SA. Accordingly, SA must be inferred by observations of team behaviors. Note that the current study presented validity evidence using video-recorded team resuscitations, and therefore it has not been validated for real time coding of events by observers who are physically present during the event. We recommend further research on that and at the current juncture we encourage the use of the team SA rating system for video-recorded resuscitations.

The dimensions of the current team SA framework and Endsley’s (1999) framework are mapped to each other in the table below. Given that observers must infer team SA through behavioral observation, some of the highly cognitive dimensions of Endsley’s facets are not captured. For example, memory accuracy and memory failure are difficult to infer through observation. However, the remaining Endsley facets are represented in at least one of the current study’s proposed team SA dimensions. Moreover, all of the proposed dimensions are captured in Endsley’s framework. Thus, it appears that the proposed taxonomy overlaps well with Endsley’s framework, lending further content validity to our model of team SA.

| **Current SA Dimensions** | **Endsley (1999) Dimensions and Facets**  **(values in parentheses refer to the current study’s proposed dimension number, “--” if there is no clear correspondence)** |
| --- | --- |
| 1. Allocate Resources  2. Anticipate And Plan  3. Avoid Fixation Errors  4. Call For Help When Needed  5. Prioritize Attention  6. Reassess patients  7. Shared Mental Model | Correctly Perceive Situation |
|  | - Data are available (--) - Data can be detected/perceived (1, 3, 5, 6, 7) - Scan or observe data (6)   - Omission (6)   - Attentional narrowing/distraction (3, 5)   - Task load management (1, 4, 5) - Accurate perception of data (3, 7) - Accurate memory (--) |
|  | Comprehend Situation |
|  | - Shared mental model (6, 7) - Accurate mental model (1, 2, 3, 7) - Appropriate use of default mental model (2, 3, 6, 7) |
|  | - Memory failure (--) |
|  | Project The Situation Into The Future |
|  | - Shared mental model (2, 7) - Accurate mental model (1, 2, 3, 5, 7) |

Source:

Endsley MR A taxonomy of situation awareness errors. In Fuller R, Johnston N, and McDonald N editors. Human Factors in Aviation Operations Aldershot, England: Avebury Aviation, Ashgate Publishing Ltd; 1999. p 287-292.
